# Deformation characteristics and motion process prediction analysis of the Lanbazi landslide in Wanzhou District, Chongqing

**DOI:** 10.1038/s41598-025-18707-2

**Published:** 2025-10-06

**Authors:** Hua Xue, Zhenwei Dai, Weizhi Jiao, Weibing Qin, Shi Cheng, Xingxing Zhao, Qihui Xiong

**Affiliations:** 1 College of Civil Engineering & Architecture, China Three Gorges University, Yichang, 443002 China; 2 Wuhan Center, China Geological Survey (Geosciences Innovation Center of Central South China), Wuhan, 430205 China; 3 Faculty of Engineering, China University of Geosciences, Wuhan, 430074 China; 4 Hubei Key Laboratory of Operation Safety of High Dam and Large Reservoir, Yichang, 443133 China; 5 Chongqing Bureau of Geology and Mineral Resources Exploration and Development Nanjiang Hydrogerlogy Engineering Geology Team, Chongqing, 401121 China

**Keywords:** SBAS-InSAR, RAMMS numerical simulation, Motion process prediction analysis, Lanbazi landslide, Engineering, Environmental sciences, Natural hazards

## Abstract

The Lanbazi landslide, a typical reservoir landslide in the Three Gorges Reservoir, has exhibited significant and increasing deformation over the past two years, posing a severe threat to the safety of nearby residents’ lives and property. This study employed a combination of field investigation, engineering geological survey, SBAS-InSAR interpretation, and RAMMS numerical simulation to predict and analyze the spatial and temporal evolution of landslide deformation and the instability movement of the Lanbazi landslide. The results suggest that the deformation rate of the landslide ranges from − 73.5 mm/a to 24.7 mm/a from January 2022 to December 2024, and the deformation of the middle and rear edge of the landslide is the largest and the movement rate is the most significant. The RAMMS software is used to calculate the movement process of the secondary potential landslide instability area. The total time from the start to the end of the landslide is 275 s, the maximum movement speed is 25.2 m/s, the maximum movement accumulation height is 31 m, the maximum impact force is 1265.2 kPa, and the landslide accumulation body will eventually flow into the Yangtze River, which will produce a surge of up to 11.7 m. This study innovatively combines SBAS-InSAR and RAMMS numerical simulation technology to realize the collaborative analysis of landslide deformation monitoring and instability motion prediction. This method breaks through the separation problem of deformation analysis and disaster prediction in traditional research.

## Introduction

The Three Gorges Hydropower Station is the largest hydropower station in the world and one of the largest engineering projects ever constructed in China. Since the reservoir began to be impounded in 2003^1^ ,more than 5,000 landslides^2–4^ or potential landslides have been identified in the Three Gorges Reservoir Area, posing a severe threat to the life and property safety of local residents and the operation of the Three Gorges Project. Therefore, it is urgent to analyze the deformation and failure motion process of the reservoir bank landslides and to provide a scientific basis for the prevention and control of landslide disasters.

At present, there have been numerous studies on both landslide monitoring and the analysis of their motion processes. Traditional landslide monitoring methods, such as mapping and hydrogeological methods^5^can obtain some information about landslides to a certain extent, but they have limitations such as limited monitoring range, low spatiotemporal resolution, high human and material costs, and the inability to capture subtle landslide deformations. In recent years, with the rapid development of remote sensing technology, synthetic aperture radar interferometry (InSAR)^6^ as an emerging technology, has become one of the most practical and effective technologies in the field of landslide monitoring due to its advantages of all-weather, all-time, large-area monitoring, and high-precision acquisition of surface deformation information.

In 1996, Achache et al. first applied InSAR to landslide monitoring^7^. Among these techniques, the small baseline subset synthetic aperture radar interferometry (SBAS-InSAR) technique, by combining multiple SAR images, effectively overcomes the problems of temporal decorrelation and atmospheric delay, and can monitor the subtle deformation of the landslide body over a long period and with high precision, providing strong data support for in-depth studies of landslide deformation evolution laws.Guo, C. et al.^8^ used the SBAS-InSAR method combined with remote sensing interpretation to obtain the surface deformation characteristics of the Xiongba ancient landslide on the west bank of the Jinsha River and found that the Xiongba ancient landslide is currently undergoing continuous creep sliding. Tian, H.B. et al.^9^ used Sentinel-1 A satellite images and applied the SBAS-InSAR method to quantify surface subsidence on the Loess Plateau and inferred landslide deformation rates. Li, Y. et al.^10^ used the SBAS-InSAR technique to obtain surface deformation and analyzed the line-of-sight (LOS) direction deformation characteristics of the landslide and its relationship with precipitation, and performed two-dimensional/three-dimensional (2D/3D) deformation decomposition to reveal its motion characteristics. Wei, Z. et al.^11^ used SBAS-InSAR and Sentinel-1 A satellite data to accurately identify active landslide areas, analyze landslide deformation trends, and found that landslide deformation is closely related to rainfall. Jiao, W. et al.^12^ combined field surveys, InSAR monitoring, and numerical simulations to conclude that the Shuiyunshan landslide is mainly driven by the effective rainfall accumulation in the rear concave catchment area and the continuous seepage and erosion of the rear reservoir.

The research methods for predicting the motion process of landslides mainly include physical simulation, numerical simulation, or a combination of both^13–16^. Wei, Gang. et al.^17^ focused on the Hongyacun landslide cluster in Qinghai Province, China, and employed advanced numerical modeling to reconstruct the full kinematic process of four sub-landslides. Zhao, Zhou. et al.^15^ took the Street landslide in Ningqiang County, Shaanxi Province, China as an example and employed the discrete element method to predict the dynamic process of the landslide and its damage to brick-concrete structure buildings. Ma, Hangsheng. et al.^18^ proposed a two-way coupled discrete element method (DEM) and smoothed particle hydrodynamics (SPH) method to study the whole hazard chain of LGIWs in mountain reservoirs. Huang, Yuezu. et al.^13^ employed the Particle Flow Code (PFC) to simulate the potential failure process of the landslide based on the three-dimensional numerical model according to the geological features and the micro-parameters. Di, Y. et al.^19^ determined the parameters of the landslide through laboratory tests and the RAMMS numerical simulation software and analyzed the risk of the landslide dam. The runout process is revealed through a combined analysis of RAMMS numerical simulations and landquake signal inversion by Liu, Y^20^.

Although most researchers have either used InSAR alone to investigate surface deformation characteristics or relied solely on numerical simulations to study landslide kinematics, systematic research that integrates deformation features with motion-process prediction remains relatively rare. Taking the Lanbazi landslide as an example, this study aims to explore the methods and techniques of the joint application of SBAS-InSAR and RAMMS in depth, to build a complete system for landslide deformation monitoring and motion process prediction, to provide new ideas and technical means for the scientific prevention and control of landslide disasters, and to play a crucial role in the early warning, risk assessment, and scientific prevention and control of landslide disasters.

## Study area

### Geological setting

Wanzhou District of Chongqing is located in the middle and upper reaches of the Yangtze River, approximately 280 km upstream of the Three Gorges Dam^21^, with geographical coordinates ranging from 107°55′22″E to 108°53′25″E and 30°24′25″N to 31°14′58″N. Wanzhou District has a subtropical monsoon humid climate with abundant rainfall. The average number of rainy days per year is around 140, with an average annual rainfall of 1191.3 mm. The majority of the rainfall is concentrated from May to September, often in the form of heavy rain, with daily rainfall exceeding 100 mm. Due to its proximity to the Three Gorges Dam, Wanzhou District has experienced frequent landslide disasters in recent years^22–25^.

**Fig. 1:**
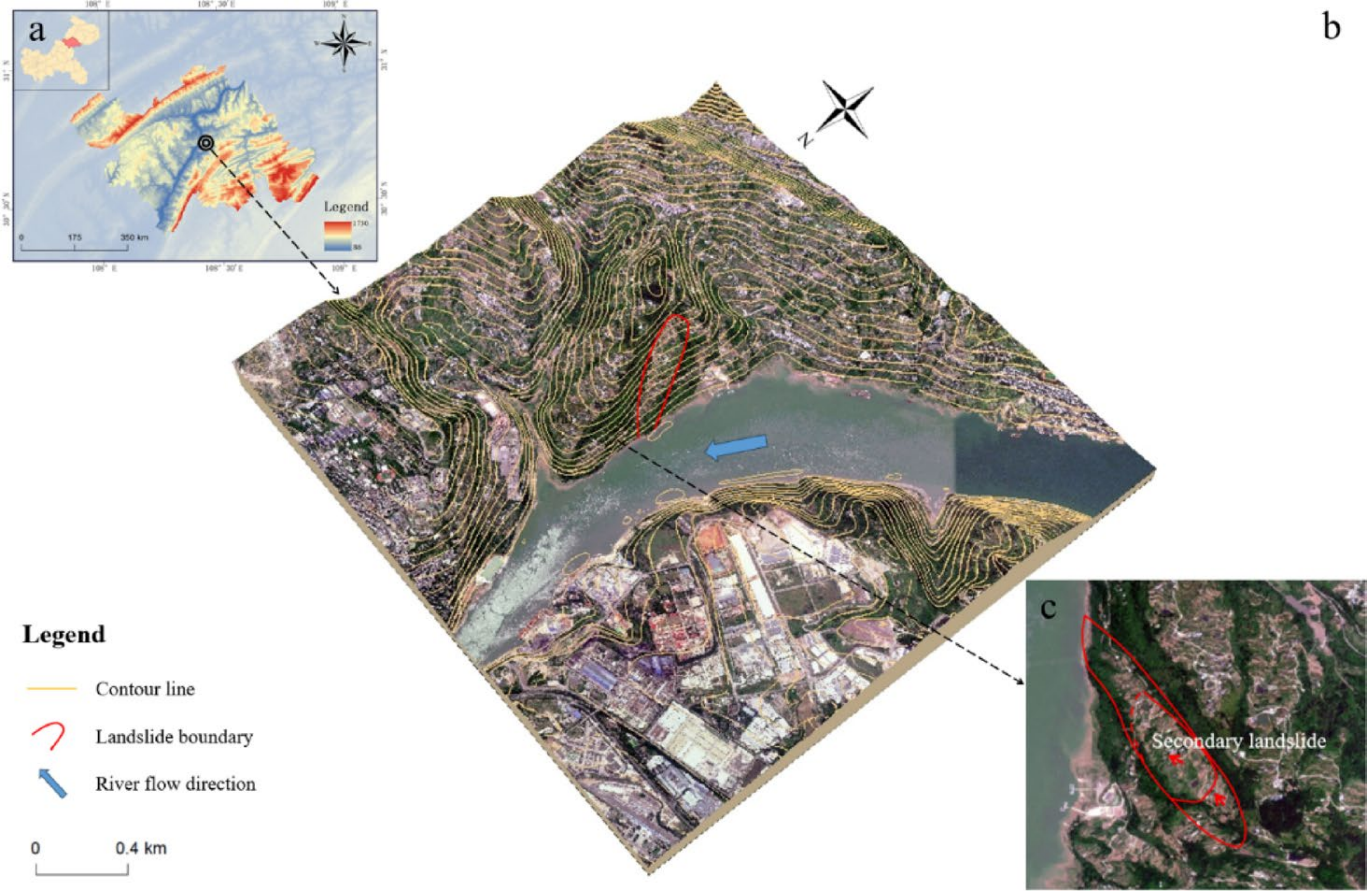
(**a**) Geographical location of Wanzhou District; (**b**) Three dimensional topographic map of the research area; (**c**) Range of the Lanbazi landslide.

The Lanbazi landslide (Fig. 1) is situated in Tanshao Village, Xintian Town, Wanzhou District, approximately 4 km from the urban area of Xintian Town and 15 km from the main urban area of Wanzhou. The landslide is located in the secondary hilly area of the northwestern wing of the Fangdou Mountain anticline, with its base adjacent to the right bank of the Yangtze River. The terrain is controlled by geological structures and generally slopes toward the northwest, forming a single-dip slope. The regional micro-topography is dominated by slopes with gradients generally ranging from 10° to 35°, with an average slope angle of about 15°. There are local gentle slope platforms. Steep cliffs and slopes are formed by thick sandstone in the reverse and tangential slope areas. The landslide body and the affected area include 71 households with 238 residents., all of whom are local original inhabitants and some resettled immigrants from the reservoir.

### General landslide feature

**Fig. 2:**
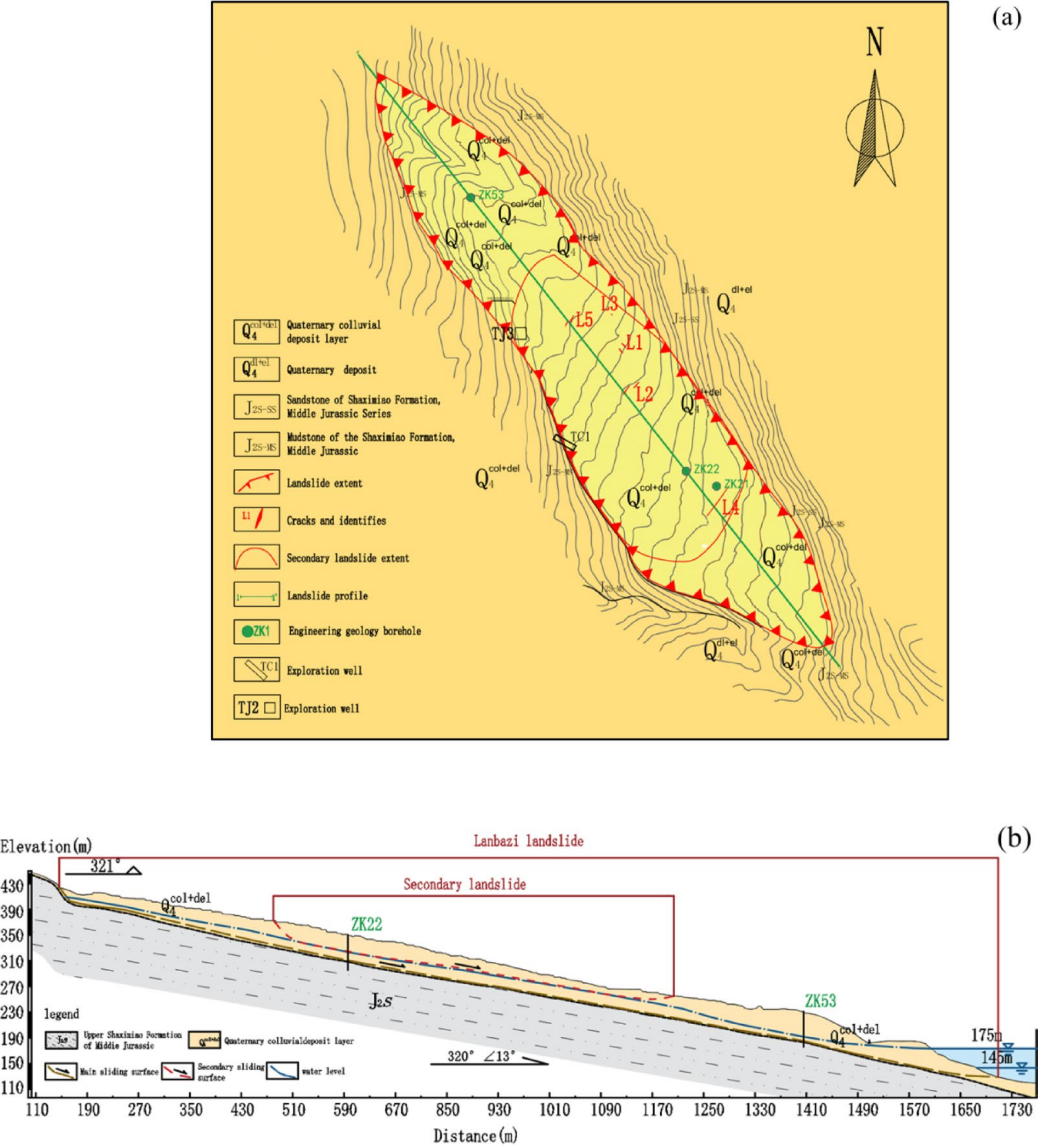
(**a**) Plan view; (**b**) geological profile of the Lanbazi landslide.

The Lanbazi landslide is located on the right bank of the Yangtze River. It has an overall elongated planform shape, with a length of about 1450 m and a width ranging from 200 to 300 m. The area is approximately 32 × 10^4^ m^2^, the average thickness of the slide mass is about 31 m, and the volume is about 990 × 10^4^ m^3^. It is classified as a deep-seated large-scale soil mass bank landslide. The left boundary of the landslide is at the top of the free-face rock slope, and the right boundary is at the foot of the steep cliff. The overall potential main sliding direction of the landslide is 320°(Fig. 2b). The front edge of the landslide is located in the water level fluctuation zone and the inundation area of the reservoir, while the rear edge is at the lower part of the rock slope with an elevation of 425 m. The relative relief of the landslide is about 296 m. The landslide materials consist of silty clay with gravel and blocky soil. The silty clay with gravel is located in the upper-middle part of the soil layer, and the blocky soil is in the lower-middle part. The soil layer is thicker near the north-east side and becomes thinner towards the south-west side. The soil layer structure is relatively dense.

The secondary landslide has a planform shape that is slightly “tongue”-shaped. Its left boundary is at the top of the rock cliff and steep slope, and its right boundary is basically consistent with the boundary of the entire landslide. The right boundary in the middle and rear part is at the foot of the rock cliff, and the front part is within a concave terrain. The relative relief of the ground in the secondary landslide area is 141 m. The width of the landslide ranges from 180 to 300 m, and the length is 570 m. The area is about 13.9 × 10^4^ m^2^, the average thickness is about 16.5 m, and the volume is about 230 × 10^4^ m^3^. It is classified as a large-scale deformation body of medium depth. The potential sliding surface is at the soil-rock contact surface in the left front part, and the main sliding direction is 300°. The materials of the secondary landslide mainly consist of silty clay with gravel, which is distributed in the middle part of the landslide and in the upper-middle part of the soil layer. The thickness of the slide mass is larger in the middle and rear part of the landslide area and thinner in the front part, with a thickness ranging from 8.1 to 27.0 m and an average thickness of about 16.5 m.

The landslide area is underlain predominantly by Holocene colluvial-slide deposits (Q4^col + del^), with minor construction fill (Q4^ml^) in the residential zone. Along the Yangtze River channel at the landslide toe, fluvial-alluvial (Q4^al^) sandy gravel beds are present. Small patches of residual-slope and colluvial deposits occur around the periphery of the landslide zone. The regional bedrock consists of Middle Jurassic Shaximiao Formation (J_2_S) mudstone and sandstone.

**Fig. 3:**
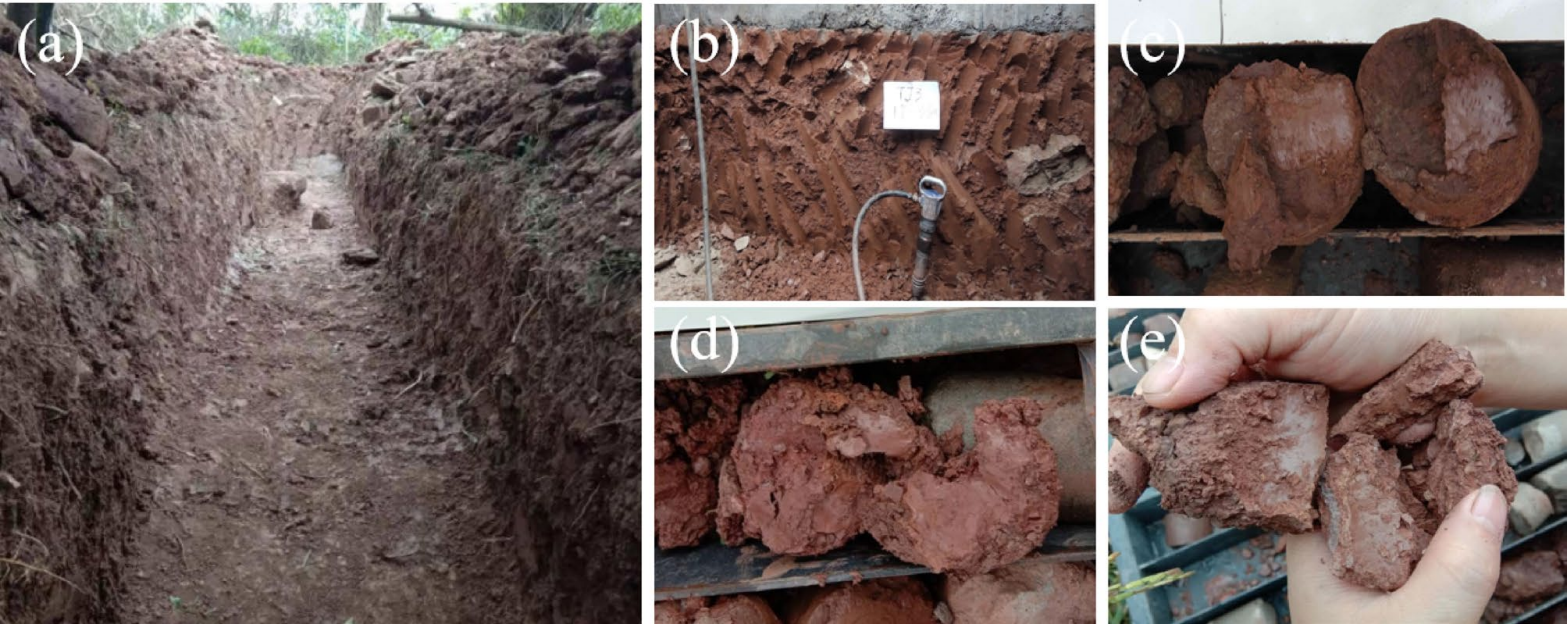
(**a**) Trench TC1; (**b**) Exploration well TJ3; (**c**) The slip surface at the depth of 44.9 m in borehole ZK53 ; (**d**) The slip surface at the depth of 25.5 m in borehole ZK22; (**e**) The slip surface at the depth of 39.4 m in borehole ZK21.

According to the exposure from trenches (Fig. 3a), exploration well (Fig. 3b) and boreholes (Fig. 3c), there are two sliding zones in the landslide, namely the shallow and deep sliding zones. The secondary landslide surface is located in the middle and rear edge of the landslide, with an average depth of about 25.5 m (Fig. 3d). The sliding zone material is silty clay with gravel. This sliding zone forms a secondary landslide area, which has a planform shape of “tongue”. Its left and right boundaries are basically coincident with the boundary of the entire landslide. The width of the landslide ranges from 180 to 300 m, the length is 570 m, the area is about 13.9 × 10^4^ m^2^, the average thickness of the secondary landslide mass is about 16.50 m, the volume is about 230 × 10^4^ m^3^, and the relative relief is 141 m. The deep sliding zone is located at the interface between the Quaternary collapse and slide accumulation layer (Q₄col + del) and the underlying middle Jurassic Shaximiao Formation sandstone and mudstone (J₂s) bedrock. The average depth of the deep sliding zone is about 39.4 m (Fig. 3e). The sliding zone material is gravelly silty clay and blocky soil. When the reservoir water level is at a high level of 175 m, the entire deep sliding zone is below the groundwater level, and part of the shallow sliding zone in the middle and rear edge of the landslide is also below the groundwater level.

## Methods

### Field investigation and engineering exploration

Multiple field investigations were conducted on the landslide. Drones were used to capture aerial images to identify the boundary morphology and macroscopic surface deformation characteristics of the landslide. Engineering exploration was carried out to determine the structural composition of the landslide, including the material composition and development depth of the landslide mass, sliding zone, and sliding bed, as well as the distribution of groundwater. Deformation cracks in the ground and building walls were also surveyed.

### SBAS-InSAR interpretation

The Small Baseline Subset Interferometric Synthetic Aperture Radar (SBAS-InSAR) technique^26–29^ is a time-series InSAR^30–33^ method that has been developed based on Differential Interferometric SAR (D-InSAR)^34–36^ and Permanent Scatterer Interferometry SAR (PS-InSAR)^37–39^ method that has been developed based on Differential Interferometric SAR (D-InSAR). This method is based on the stable and reliable phase information from high-coherence scatterers and uses iterative phase model fitting to estimate and remove various error terms in order to extract subtle deformation signals. During the computation, a time-series analysis of a large number of SAR images accumulated over the deformation area effectively separates the subtle deformation phase from orbital and atmospheric error phases, thereby achieving high-precision measurement of crustal deformation along fault zones. SBAS-InSAR is a multi-master image-based time-series InSAR technique that requires a relatively limited data and overcomes the effects of temporal-spatial baseline decorrelation and atmospheric influences, providing continuous deformation information in both time and space.

**Fig. 4:**
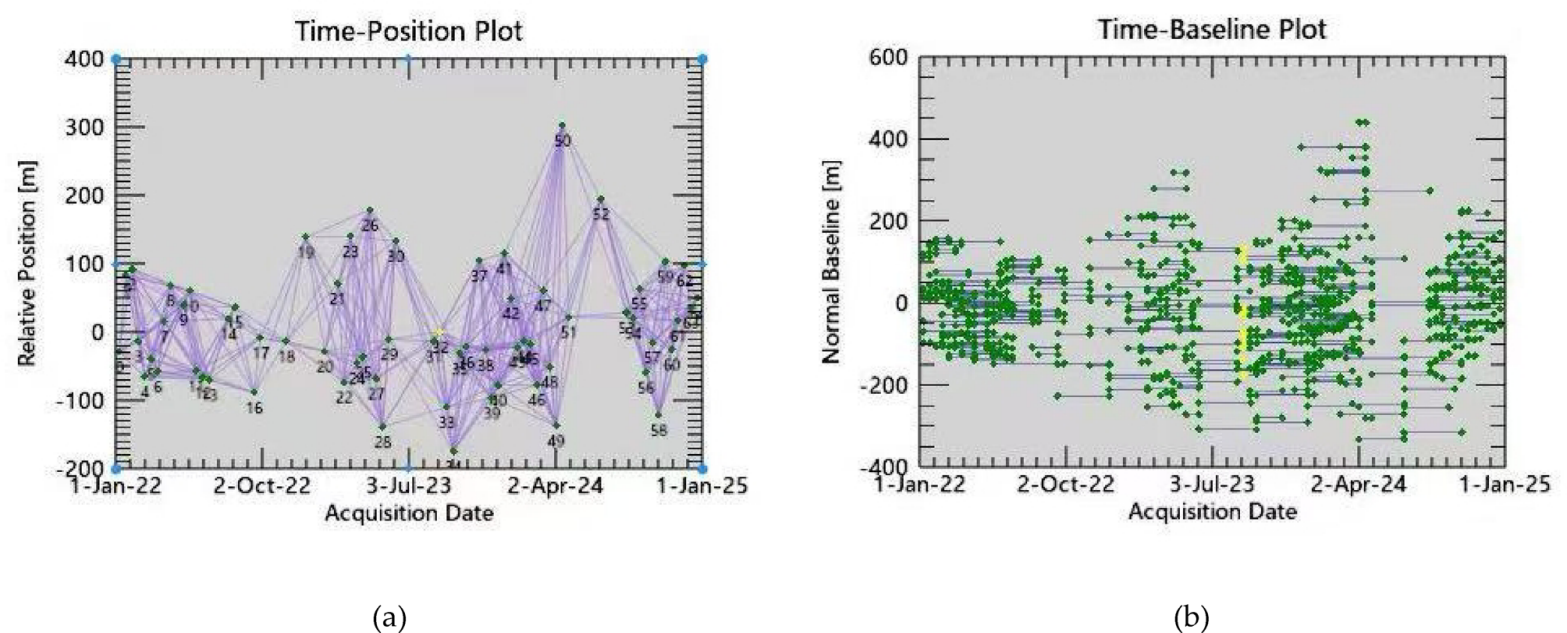
(**a**) Connection space baseline diagram; (**b**) Connection time baseline diagram.

In this study, 65 ascending track images from Sentinel-1 A, acquired from January 2022 to December 2024, were selected. To ensure effective interferometric pairs, the temporal baseline threshold was set to 120 days, and the spatial baseline threshold was set to 45%, resulting in the generation of 265 interferometric pairs. During data processing, a 30-meter resolution SRTM DEM (Digital Elevation Model) was used for orbit refinement and re-flattening to eliminate or reduce the impact of terrain phase. The date of the ascending master image was August 30, 2023. The spatial-temporal baseline conditions for the ascending track are shown in Fig. 4.

### RAMMS numerical simulation

The RAMMS software^40–44^, developed by the Swiss Federal Institute for Snow and Avalanche Research, is primarily used to simulate the entire process of avalanches, rockfalls, debris flows, and shallow landslides from initiation to deposition on three-dimensional terrain. The DEBRIS-FLOW module within the software is capable of predicting the spatial distribution of data such as the movement path, flow velocity, flow depth, and pressure of landslides, providing a good numerical simulation of the landslide motion state.

Utilizing the numerical simulation software RAMMS, the movement parameters of a landslide mass under three-dimensional terrain conditions, including the movement distance, velocity, impact pressure, and flow height of the landslide mass, can be rapidly simulated, and offers the advantages of simple operation, short computation time, and minimal input parameters^45–48^. In RAMMS, the Voellmy friction law, based on the Voellmy-Salm method, is employed, and the Voellmy friction model has been widely applied in mass movement simulations. The Voellmy model takes into account the resistance of both the solid phase and the viscous turbulent fluid phase, with the frictional resistance divided into two components, namely dry Coulomb friction and viscous turbulent friction. The calculation of frictional resistance is as follows (Salm 1993):

1 $$S=\mu N+\frac{{\rho g{u^2}}}{\xi }$$

2 $$N=\rho hg\cos (\varphi )$$

In the formula, S represents the frictional resistance (Pa), N is the normal stress (Pa), µ is the dry Coulomb friction coefficient, ε is the viscous turbulence coefficient, ρ is the material density of the landslide mass (kg/m^3^), g is the acceleration due to gravity (m/s^2^), φ is the slope angle (°), h is the flow height (m), and u is the velocity (m/s). The formula ensures that when the normal stress N and the vector velocity U approach zero, the frictional resistance S also approaches zero.

In the RAMMS software, the numerical model is primarily established through a digital elevation model (DEM). The accuracy of the simulation results depends on the accuracy of the input topographic data. In this study, a DJI drone was used to conduct aerial surveys of the Lanbazi landslide area, and the Pix4D software was employed for route planning, generating a high-precision DEM and orthophoto map of the landslide area.

Frictional parameters (µ and ξ) are key factors controlling the runout distance and deposition range. The RAMMS model achieves automatic matching of frictional parameter values by setting up the terrain model and release area, in combination with the terrain conditions within the program. Based on return periods, terrain characteristics, slide release volumes, and height restrictions, µ and ξ values are automatically obtained from the cell height, slope, and curvature. Subsequently, µ and ξ values that vary with the terrain are generated and saved in ASCII text format. The RAMMS software user manual summarizes landslide and debris flow cases from around the world and provides recommended ranges for µ and ξ, with µ ranging from 0.05 to 0.4 and ξ ranging from 100 to 200 m/s^2^.

The simulation is based on DEM digital elevation data with a precision of 8 m. The DEM data is converted into ASCII format and imported into the RAMMS software. After the terrain modeling is completed, the release area and parameters are determined, and the simulation time step is set to 5 s (Table 1).

**Table 1:** Parameter settings of RAMMS.

Category	Parameter	Value
Digital terrain model	Simulation grid resolution (DEM)	8.0 m
Release zone	Area	12.6 × 10^4^m^2^
	Release depth	18.50 m
	Release volume	234 × 10^4^m^3^
Cohesion	Cohesion	23.9kpa
Dump interval	Dump interval	5.0s
Friction MU XI	µ	0.2
	ξ	200 m/s^2^

## Results

### Macroscopic deformation characteristics of the landslide

Field investigations and interviews with local residents revealed that the landslide began to deform in 2017, and in recent years, deformation has intensified during rainy periods. For instance, from late September to mid-October 2017, the study area experienced continuous rainfall for over 20 days, with a cumulative rainfall exceeding 200 mm, which triggered new deformation phenomena on the landslide surface. Deformation was mainly concentrated in the secondary landslide area in the middle and rear part of the landslide and the middle front edge. The overall manifestation was tensile deformation at the rear and compressive bulging deformation at the front, with distinct characteristics of translational movement, resulting in the formation of numerous new cracks in the ground and structures.

**Fig. 5:**
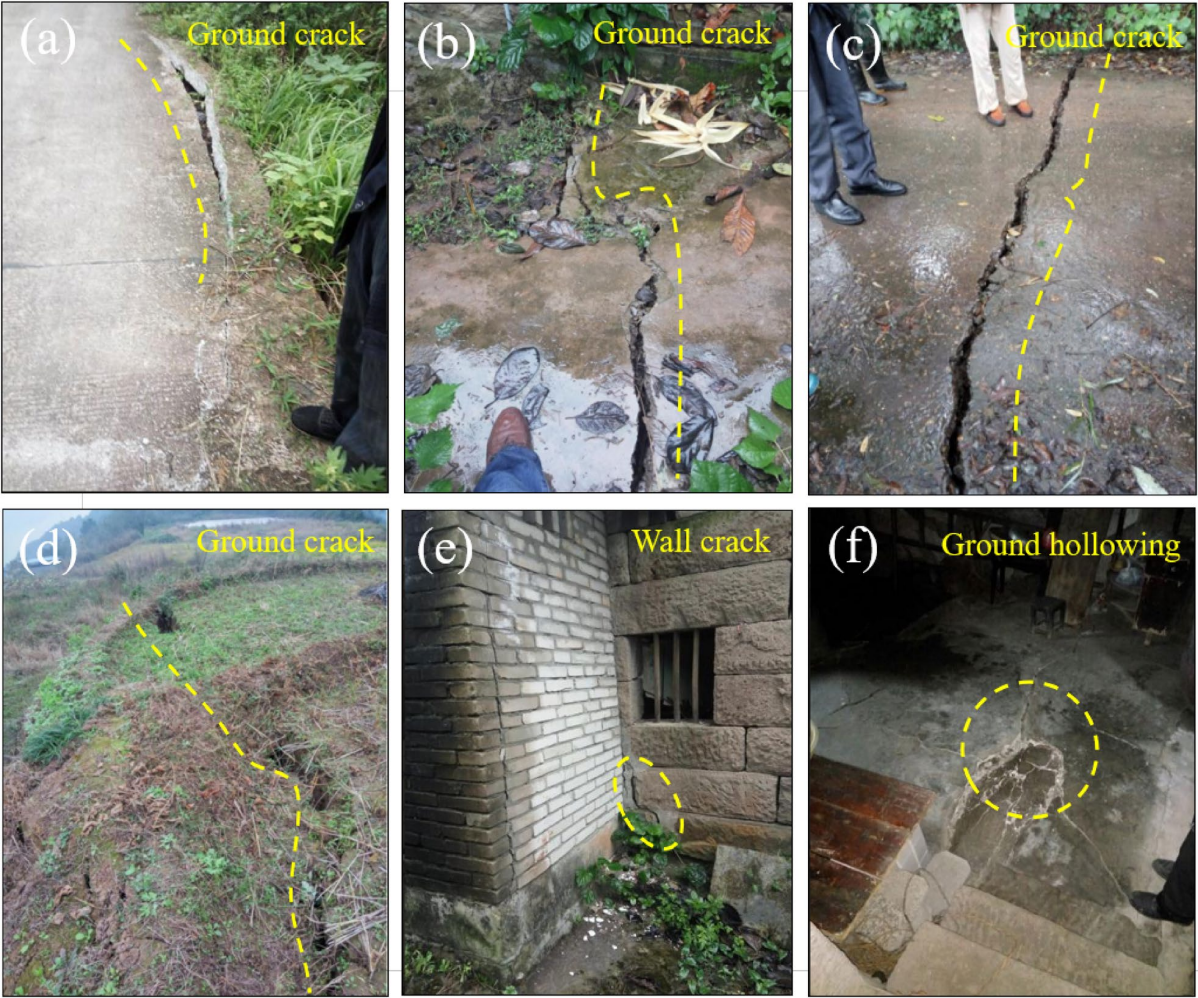
Local deformation on the surface of Lanbazi landslide.

The crack L1 depicted in Fig. 5a is located on the right side of the village road in the middle front part of the secondary landslide, with an extension length of about 20 m. The road surface exhibits segmental compression and local uplift ranging from 2 to 5 centimeters, accompanied by partial collapse The crack L2 in Fig. 5b is situated in the housing area in the middle of the secondary landslide, trending at 215°, with an extension length of 3–12 m, an opening of 1–3 cm, and a visible depth of 2–5 cm. The crack L3 in Fig. 5c is located on the right side of the village road in the middle of the secondary landslide, trending at 130°, with an extension length of about 8 m and an opening of 2–3 cm. The crack L4 in Fig. 5d is at the rear of the secondary landslide, trending at 212° and dipping at 302°, with an extension length of about 60 m and a visible depth of 10–40 cm. It is a tensile crack with an opening of 2–40 cm, and some parts have been destroyed due to cultivation. The cracks L5 in Fig. 5e and f are in the front housing area of the secondary landslide, with ground and wall fractures trending at 213°, an extension length of about 20 m, an opening of 1–3 cm, and a visible depth of 1–3 cm. Inside the houses, there is floor bulging with an uplift of 1–5 cm. Based on the above investigation, it is evident that the long-term continuous deformation of the landslide has caused significant damage to the buildings on the landslide mass. The deformation range has expanded, and the deformation has become more pronounced, posing a severe threat to the life and property safety of the residents.

### Long-term kinematic characteristics of the landslide

**Fig. 6:**
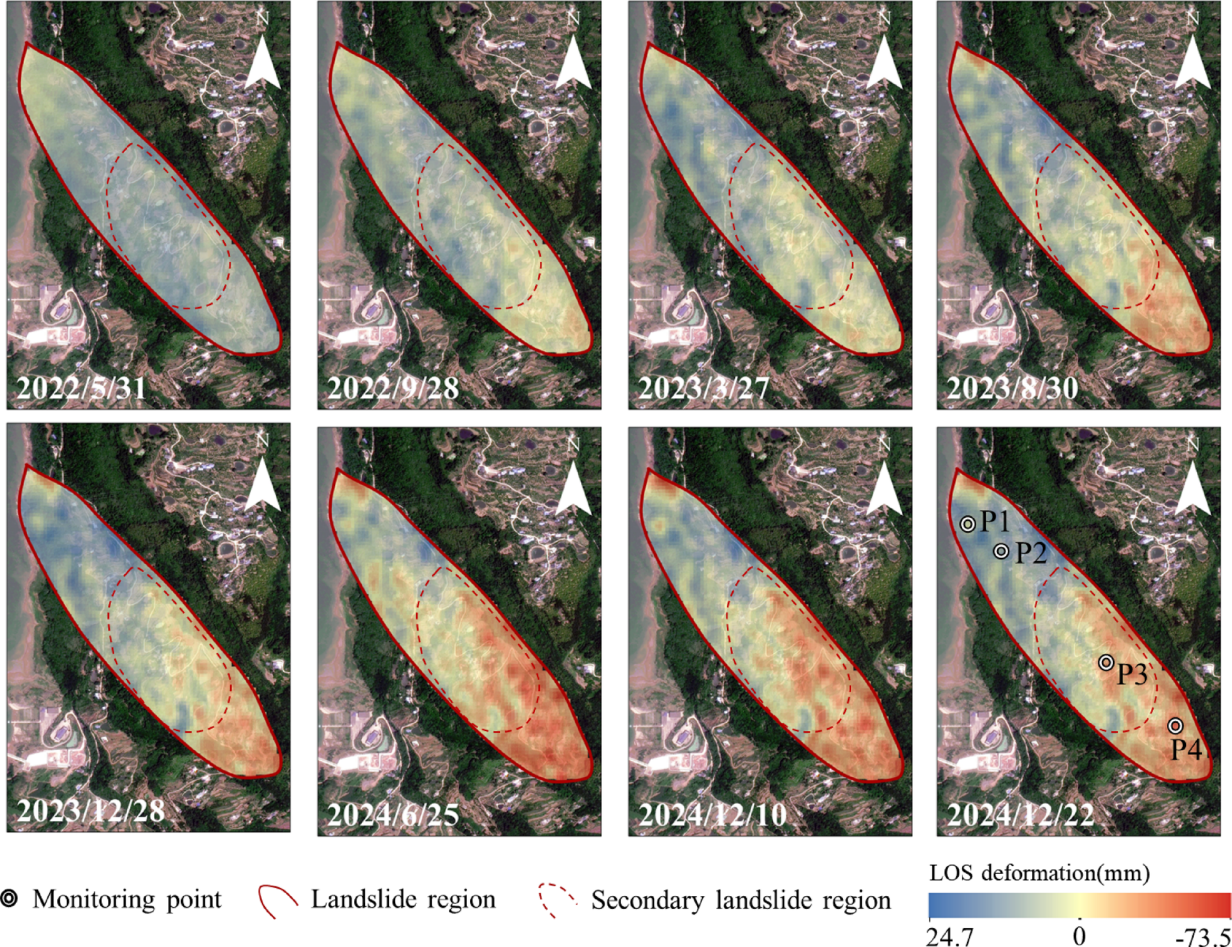
Time series deformation of Lanbazi landslide from March 2022 to December 2024.

Figure 6 presents the cumulative surface deformation time series of the landslide from 2022 to 2024, calculated using SBAS-InSAR. The results reveal that the landslide deformation exhibits significant spatial and temporal heterogeneity. From January 2023 to December 2024, the figure highlights significant surface deformation of the Lanbazi landslide, with a cumulative deformation ranging from − 73.5 to 24.7 mm/year. A positive deformation rate indicates movement of the deformation point towards the satellite, while a negative rate indicates movement away from the satellite. The figure shows that from May 31, 2022, to March 27, 2023, deformation gradually initiated from the rear edge of the landslide. From August 30, 2023, to December 28, 2023, the deformation progressively spread to the middle part, with intensified deformation at the rear. From June 25, 2024, to December 22, 2024, deformation in the middle and rear parts of the landslide continued to intensify. Overall, the deformation trend of the landslide shows a gradual decrease from the rear edge to the front edge, dominated by the rear edge and exhibiting a translational characteristic, which is significantly different from the traditional drawdown-induced failure mode of bank slope accumulation layer landslides.

**Fig. 7:**
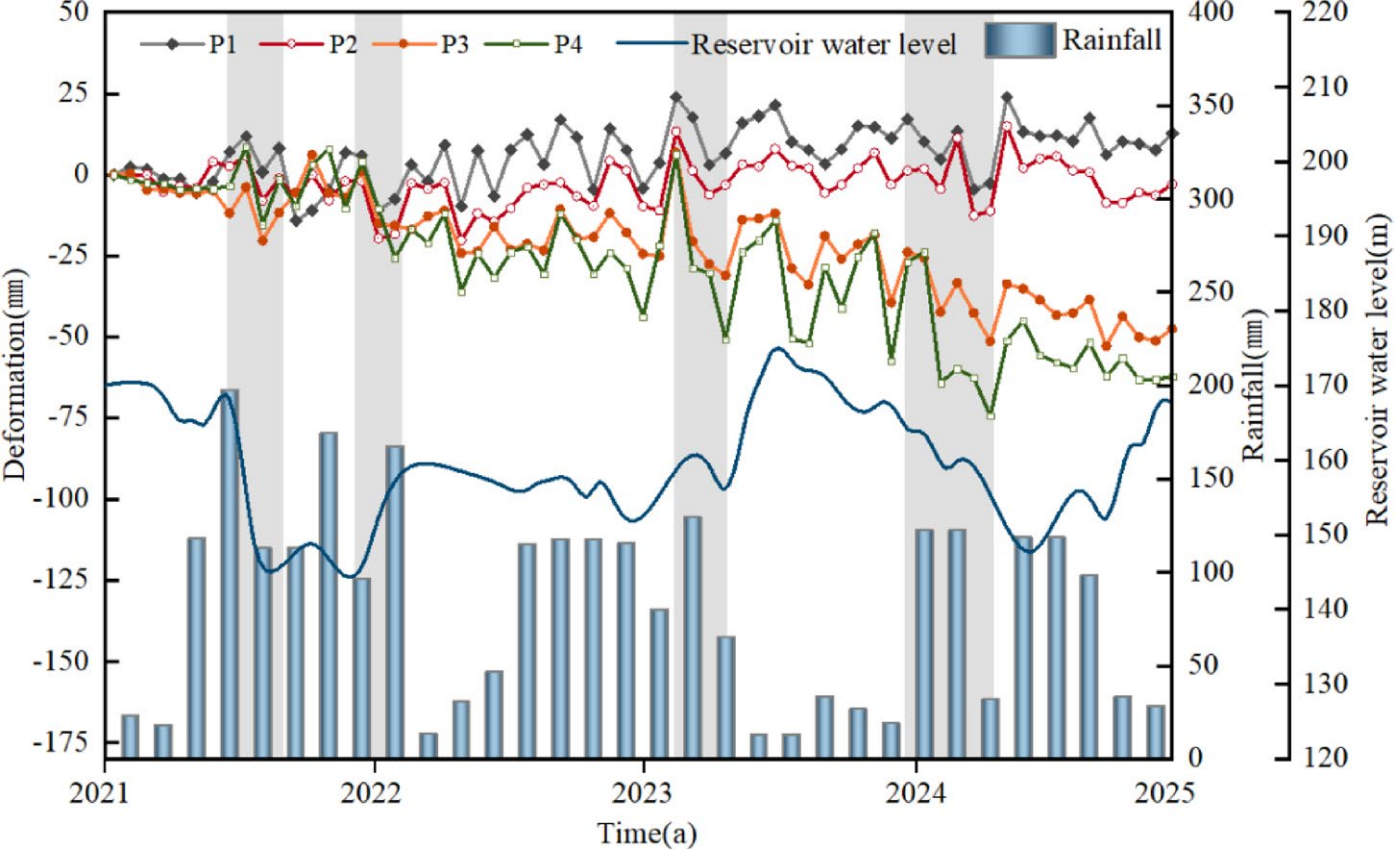
Relationship between surface cumulative deformation of landslide and rainfall.

To analyze the spatial differences in deformation, four monitoring points were strategically arranged at different locations on the landslide (Fig. 7). As can be seen from the figure, Point 4 at the rear edge of the landslide has the largest deformation, at about 60 mm; followed by Point 3 in the secondary landslide area, with a deformation of about 50 mm; while Points 1 and 2 at the front edge of the landslide have almost zero settlement. The results indicate that the movement pattern of the Lanbazi landslide is mainly characterized by overall downward sliding, with subsidence in the middle and rear parts of the landslide mass and slight uplift of the soil at the front part of the landslide mass.

The rainfall in the study area is concentrated in the summer, and the landslide is prone to accelerated deformation due to precipitation. For example, from April to May 2022, with a cumulative rainfall of 310.6 mm, the displacements of P3 and P4 increased from − 12 mm to − 3.5 mm to -20.4 mm and − 15.5 mm, representing increases of 70% and 342.9%, respectively. From September to November 2023, with a cumulative rainfall of 275.2 mm, the displacements of P3 and P4 increased from 6.8 mm to 6.3 mm to -31.1 mm and − 50.9 mm, representing increases of 357.35% and 707.94%, respectively. From April to August 2024, with a cumulative rainfall of 230.9 mm, the displacements of P3 and P4 increased from − 25.8 mm to − 23.9 mm to -51.5 mm and − 74.5 mm, representing increases of 99.6% and 211.6%, respectively.

In Fig. 7, rainfall exerts a greater influence on the landslide than reservoir water level. It is inferred that this phenomenon is due to the landslide mainly consisting of gravelly cohesive soil and gravelly soil, which have good permeability and are susceptible to accelerated deformation by precipitation. Therefore, there is a significant positive correlation between landslide deformation and monthly rainfall (Fig. 9).

### Simulation results of landslide movement process

The monitoring results from SBAS-InSAR further confirm that the secondary landslide area and the rear edge of the landslide have undergone significant deformation. Currently, the main objects threatened and affected by the landslide include 238 people, 540 houses, approximately 600 m of village-level immigrant roads at the front, about 1500 m of power lines, around 1200 fruit trees, and about 360 acres of arable land. The potential economic loss caused by the landslide is estimated to be around 40 million yuan. The objects under threat are mainly concentrated in the secondary landslide area and the affected zone, accounting for about 85% of all objects. It can be anticipated that once the landslide is rapidly activated, the secondary landslide area will be the first to suffer from landslide destruction and will be the most threatened region.

**Fig. 8:**
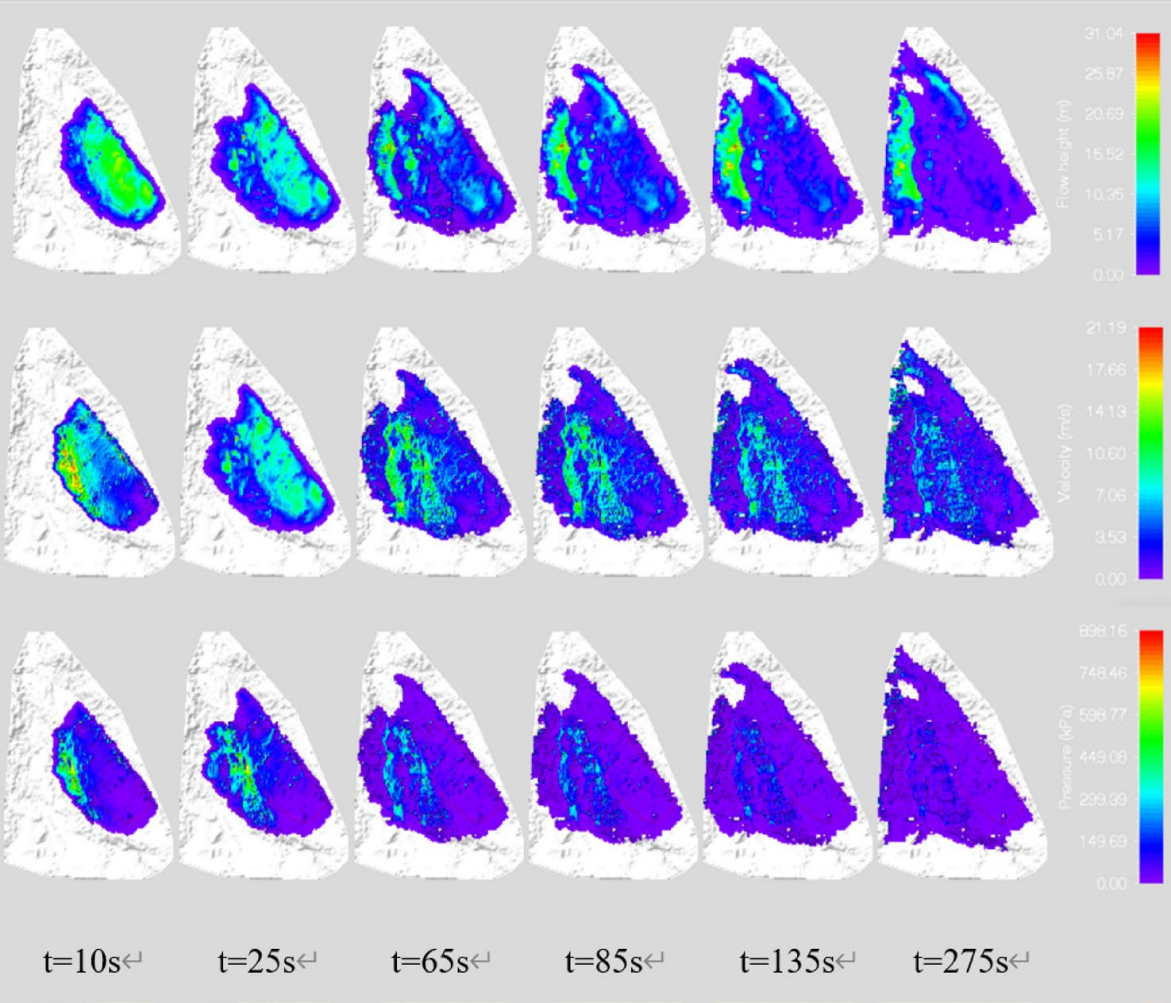
The movement outcomes of landslides at different times.

**Fig. 9:**
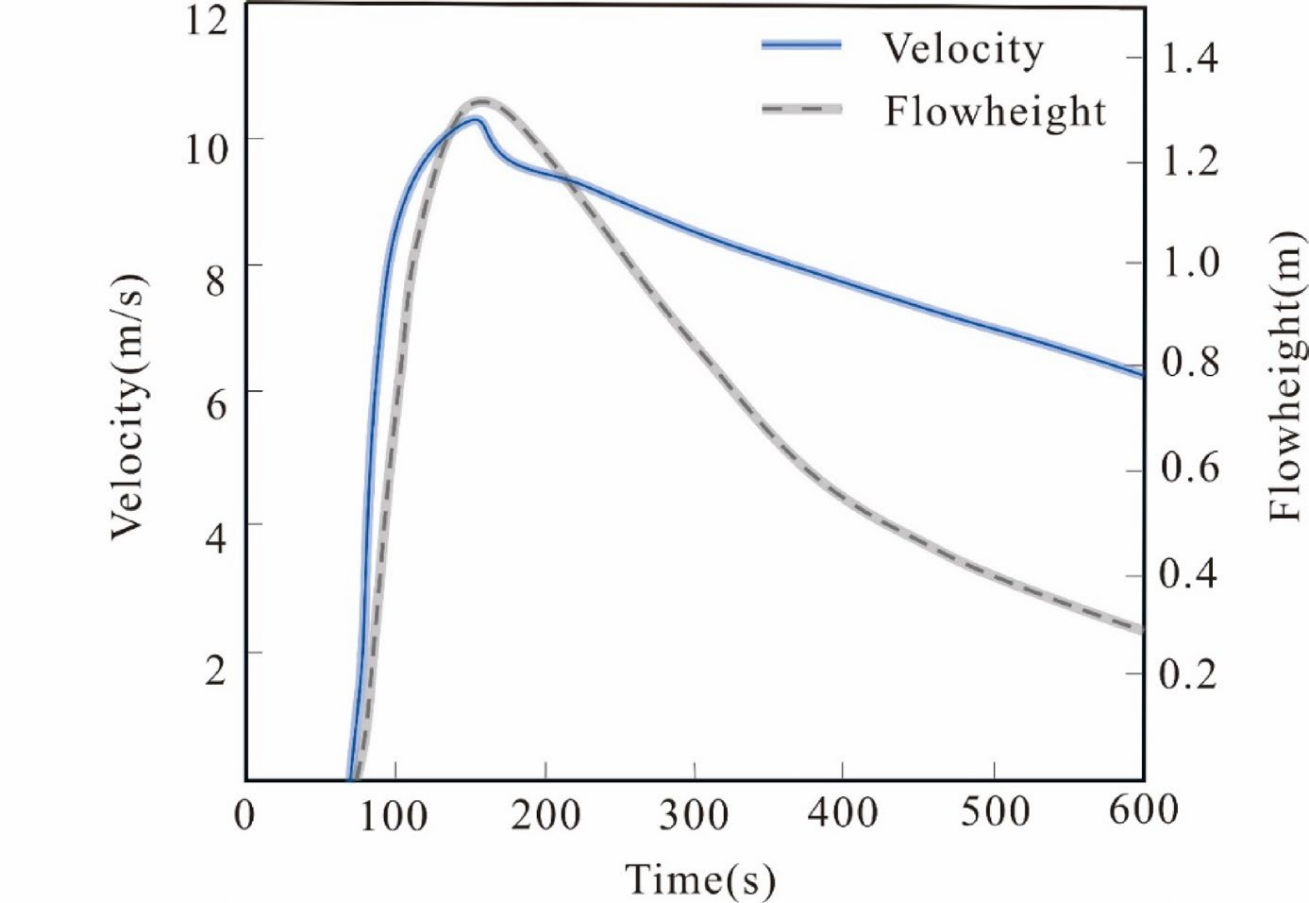
The temporal variation of landslide velocity and debris height.

To better understand the potential hazards, we conducted a predictive analysis of the movement process in the secondary landslide area. The movement height, velocity, and impact force of the landslide deposit at different moments are illustrated in Fig. 9. The total movement time from the initiation to the complete stop of the landslide is 275 s, with a maximum movement velocity of 25.2 m/s, a maximum movement deposit height of 31 m, and a maximum impact force of 1265.2 kPa. To analyze the dynamic characteristics of the landslide from initiation, movement to complete deposition and stop in detail, the movement process of the landslide was discussed at *T = 10 s*,* T = 25 s*,* T = 65 s*,* T = 85 s*,* T = 135 s*,* and T = 275 s* (Fig. 8).

After the landslide was initiated, the total duration was 275 s. Based on the simulated results of velocity changes throughout the process, the movement was divided into four stages: initiation (0–10 s), acceleration (10–25 s), deceleration (25–85 s), and deposition (85–275 s). From 0 to 10 s, the landslide began to slide along the sliding surface. Due to the steep terrain near the northeast side of the secondary landslide and the large accumulation height of the soil in the middle, the soil in this position began to accumulate rapidly, with a maximum accumulation height of 23.5 m and a maximum movement velocity of 20 m/s at this time; from 10 to 25 s, the landslide was in the acceleration stage, as the south side of the landslide area is a medium-shallow gully, the landslide began to move rapidly towards the Yangtze River direction. In addition, since the landslide is located in the secondary hilly area of the northwestern wing of the Fangdou Mountain anticline and is controlled by the structure, the terrain generally slopes towards the northwest, so the maximum accumulation height of 26.5 m and the maximum movement velocity of 21.5 m/s were reached in the northwest direction of the landslide; from 25 to 85 s, the landslide was in the deceleration stage and gradually approached the Yangtze River, with a maximum accumulation height of 30 m and a maximum movement velocity of 16 m/s.From 85 to 275 s, the movement velocity of the landslide continued to slow down. The maximum accumulation height of the landslide was 26 m, and the maximum movement velocity was 10 m/s.

However, the main secondary disaster triggered by reservoir landslides is the seiche^49–54^the hazard of which can even exceed that of the landslide disaster itself. Based on model test data from the Libby Dam and Koocanusa Lake in the United States, Slingerland and Voight’s seiche calculation method proposes a formula for calculating the maximum seiche height through a dimensionless analysis of the factors affecting landslide-induced seiches.

3 $$\:\text{log}（\frac{{\eta\:}_{max}}{h}）=-1.25+0.71\text{l}\text{o}\text{g}\left(\frac{1}{2}\frac{{\rho\:}_{s}}{{\rho\:}_{w}}\frac{{V}_{s}}{{h}^{3}}\frac{{v}_{s}^{2}}{gh}\right)$$

In the formula, η_max_ represents the maximum seiche height (m), V_s_ is the landslide volume (m^3^), h is the water depth (m), V_s_ is the landslide velocity (m/s), ρ_s_ is the landslide density (kg/m^3^), and ρ_w_ is the water density (kg/m^3^). The landslide volume is 234 × 104 m^3^, the water depth is 100 m, the landslide velocity is taken as 11 m/s (as shown in Fig. 9), the landslide density is 1.93 × 103 kg/m^3^, and the water density is 1 × 103 kg/m^3^. According to the formula, the calculated maximum seiche height is approximately 11.7 m. This not only threatens towns and residents but also affects transportation on highways, railways, and shipping, and may even impact downstream areas. Therefore, during heavy or continuous rainfall, close attention should be paid to the deformation of the landslide, especially the secondary landslide area with intense deformation and significant threats to residents. It is recommended to conduct high-frequency manual inspections for typical precursory phenomena such as crack propagation and surface uplift. If possible, it is urgent to deploy a multi-source sensor network at key locations, such as installing rain gauges, GNSS displacement meters, and crack meters, to continuously track the dynamics of the landslide.

## Conclusions

This study focuses on the Lanbazi landslide in Xintian Town, Wanzhou District, Chongqing, China, as the research object. By using field investigation, engineering geological exploration, and innovatively combined SBAS-InSAR and RAMMS numerical simulation to achieve a synergistic analysis of landslide deformation monitoring and motion-process prediction. This approach breaks the conventional separation between deformation analysis and hazard forecasting. The main conclusions are as follows:

1. The planform shape of the Lanbazi landslide is generally elongated, with a length of about 1450 m, a width of about 200–300 m, an area of about 32 × 10^4^ m^2^, an average thickness of the slide mass of about 31 m, and a volume of about 990 × 10^4^  m^3^. It is classified as a deep-seated large-scale soil mass bank landslide. Based on trench and borehole exposures, two sliding surfaces were identified: a deep sliding zone at 39.4 m and a secondary landslide area at 25.5 m.
2. The SBAS-InSAR interpretation results show that the secondary landslide area has the largest deformation. The overall cumulative deformation of the Lanbazi landslide ranges from − 73.5 to 24.7 mm/a. Based on the deformation of the four selected monitoring points, the two points in the secondary landslide area have larger deformations, approximately 50 mm and 60 mm, respectively. The two points at the front edge of the landslide have almost zero settlement. Therefore, the movement process of the secondary landslide was simulated and predicted.
3. The numerical simulation results of the movement process of the secondary landslide area show that the total release volume of the landslide is 234 × 10^4^ m^3^, the total movement time is 275 s, the maximum movement velocity is 25.2 m/s, the maximum movement deposit height is 31 m, and the maximum impact force is 1265.2 kPa. When the landslide velocity is 11 m/s, the maximum seiche height generated is approximately 11.7 m, indicating a huge potential hazard.

## Data Availability

The datasets used or analysed during the current study available from the corresponding author on reasonable request.
